# Supplementary material for: RAC1B Suppresses TGF-β-Dependent Chemokinesis and Growth Inhibition through an Autoregulatory Feed-Forward Loop Involving PAR2 and ALK5
Source: Cancers (Basel). 2019 Aug 20;11(8):1211. doi: 10.3390/cancers11081211 (PMC6721813; doi:10.3390/cancers11081211)

Supplementary Figure S1

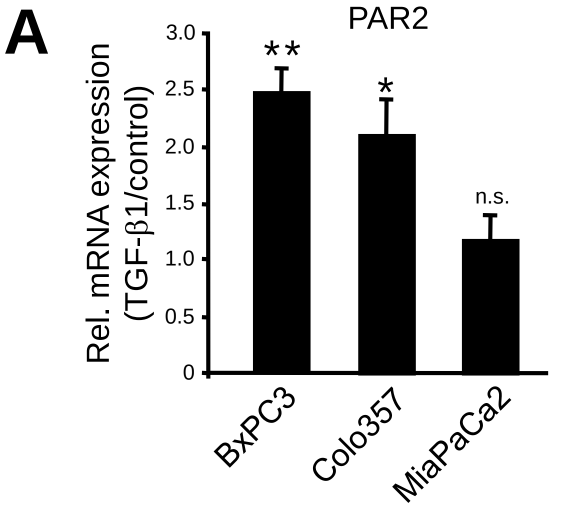

Figure S1A. Effect of TGF-β1 on PAR2 expression in various PDAC-derived cell lines. The indicated PDAC-derived cell lines were stimulated with TGF-β1 for 24 h and subsequently subjected to qPCR analysis of PAR2 and TBP. Data represent the normalized mean ± SD of three assays. The asterisk indicate significance compared to untreated controls.

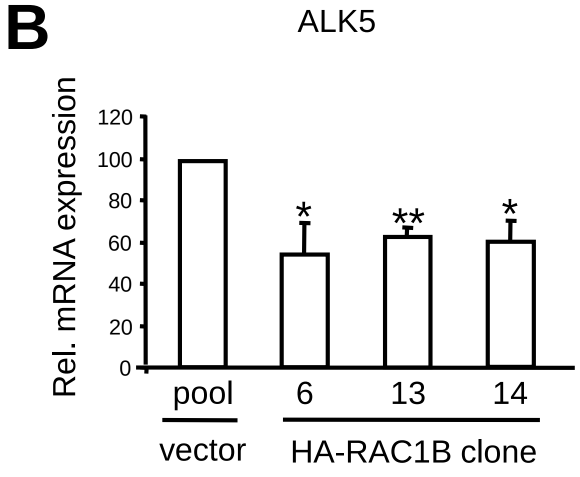

Figure S1B. Effect of stable ectopic RAC1B overexpression on ALK5 mRNA expression. Three individual clones of Panc1 cells with stable ectopic expression of HA-tagged RAC1B (HA-RAC1B) or empty vector (vector) were subjected to qPCR analysis of ALK5. Data represent the normalized mean ± SD of three parallel wells from one representative experiment out of three experiments performed in total. The asterisk indicate significance compared to the vector control.

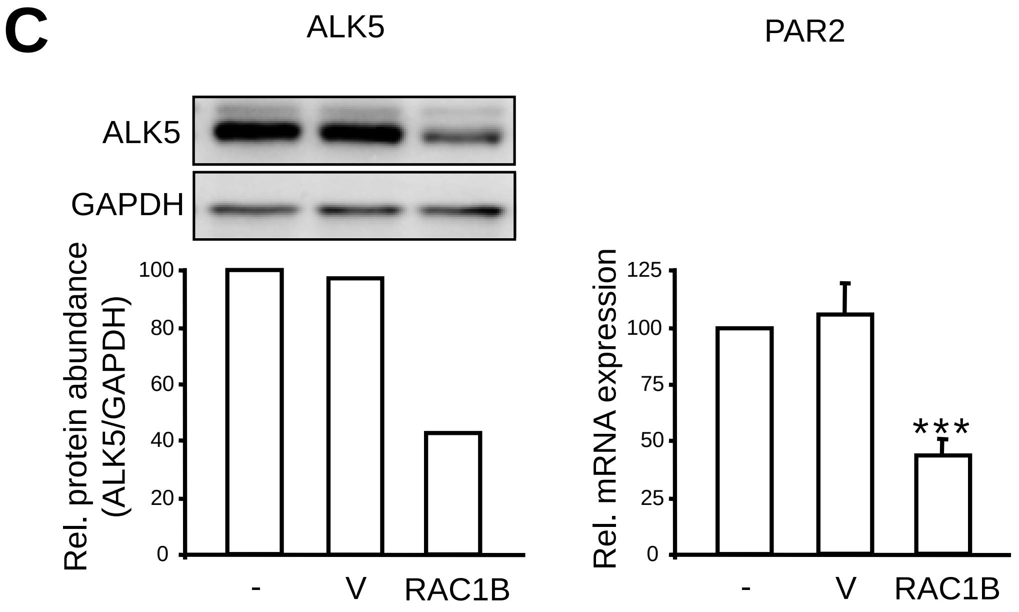

Figure S1C. Effect of transient transfection of HA-RAC1B into Panc1-RAC1B-KO cells on ALK5 protein expression and PAR2 mRNA expression. Left-hand graph, Panc1 cells mock-transfected (-), or transfected with either empty plasmid vector (V) or the same vector encoding HA-tagged RAC1B (RAC1B) were lysed 48 h after the start of transfection and subjected to immunoblot analysis of ALK5. Right-hand graph, The same as in the left graph, except that cells were subjected to RNA isolation and qPCR analysis of PAR2. Data shown are the means ± SD of three wells processed in parallel. In both graphs one of two assays is shown both with very similar results. The asterisk indicate significance compared to mock-transfected controls.

Supplementary Figure S2

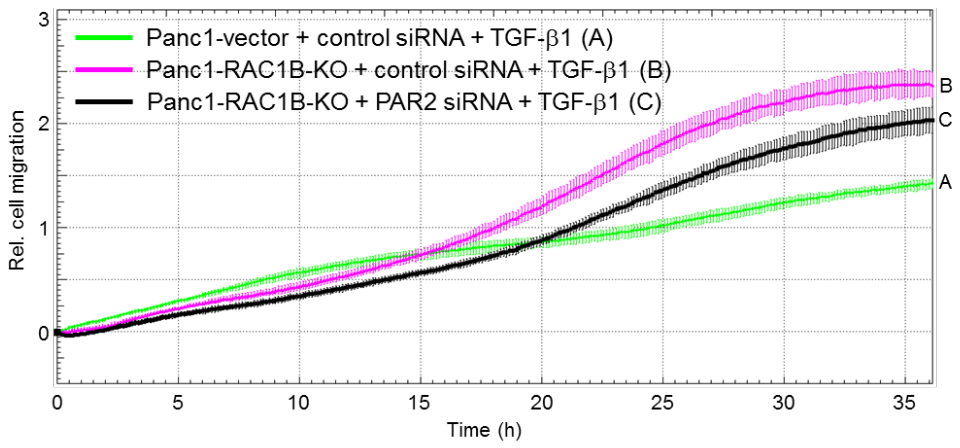

Figure S2. Effect of PAR2 knockdown on TGF-β1-induced chemokinesis in Panc1-RAC1B-KO cells. Panc1-RAC1B-KO and vector control cells were transfected twice with 50 nM of either control or PAR2 siRNA and 48 h later assayed for migratory activity on an xCELLigence platform in the presence of TGF-β1. The graph shows a representative experiment. Data are the mean ± SD from 3-4 wells per condition. Differences between Panc1-RAC1B-KO + PAR2 siRNA + TGF-β1 (black curve, tracing C) and Panc1-RAC1B-KO + control siRNA + TGF-β1 (magenta curve, tracing B) are significant at 16:30 and all later time points. Successful inhibition of RAC1B and PAR2 was verified by immunoblotting and qPCR analysis, respectively (not shown).

Supplementary Figure S3

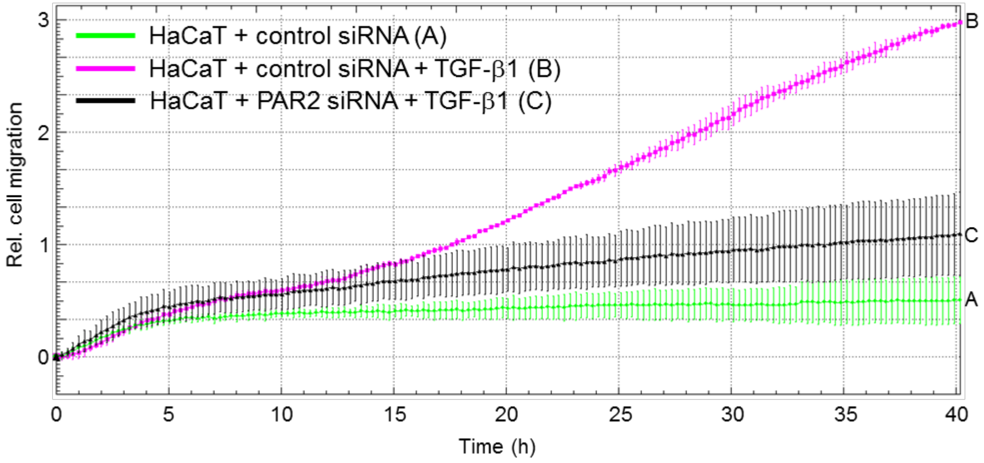

Figure S3. Effect of PAR2 knockdown on TGF-β1-induced chemokinesis in HaCaT cells. HaCaT cells were transfected twice with 50 nM of either control or PAR2 siRNA and 48 h later assayed for chemokinetic activity by RTCA technology in the absence or presence of TGF-β1. The graph shows a representative experiment. Data are the mean ± SD from 3-4 wells per condition. Differences between HaCaT cells + PAR2 siRNA + TGF-β1 (black curve, tracing C) and HaCaT cells + control siRNA + TGF-β1 (magenta curve, tracing B) are significant for the first time at 17:00 and all later time points. Successful inhibition of PAR2 was verified by qPCR analysis (data not shown).

Supplementary Figure S4

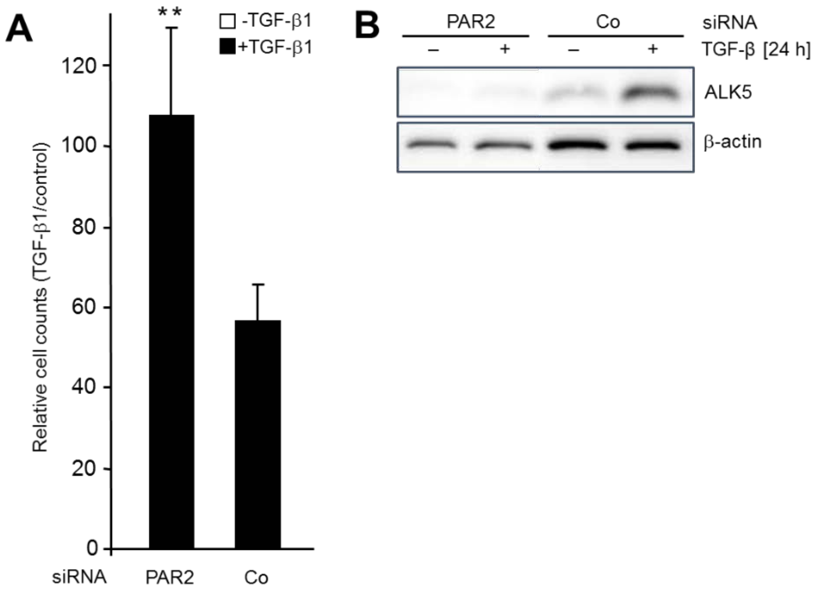

Figure S4. Effect of PAR2 knockdown on the growth-inhibitory effect of TGF-β1 in HaCaT cells. (**A**) HaCaT cells were transfected twice with 50 nM of either PAR2 siRNA or control (Co) siRNA. Forty-eight hours after the second round of transfection cells were treated, or not, with TGF-β1 for 24 h, then detached and counted. Data are the mean ± SD of three experiments and are displayed as % reduction in cell numbers of TGF-β1-treated cells relative to numbers of untreated control cells. The asterisks indicate significant difference. (**B**) As in (**A**) except that cells were lysed after TGF-β1 treatment and processed for immunoblotting of ALK5, and β-actin as a loading control.

Supplementary Material: Full blots and band quantification: Figure 1C and Supplementary Figure S1C

Immunoblot Figure 1C

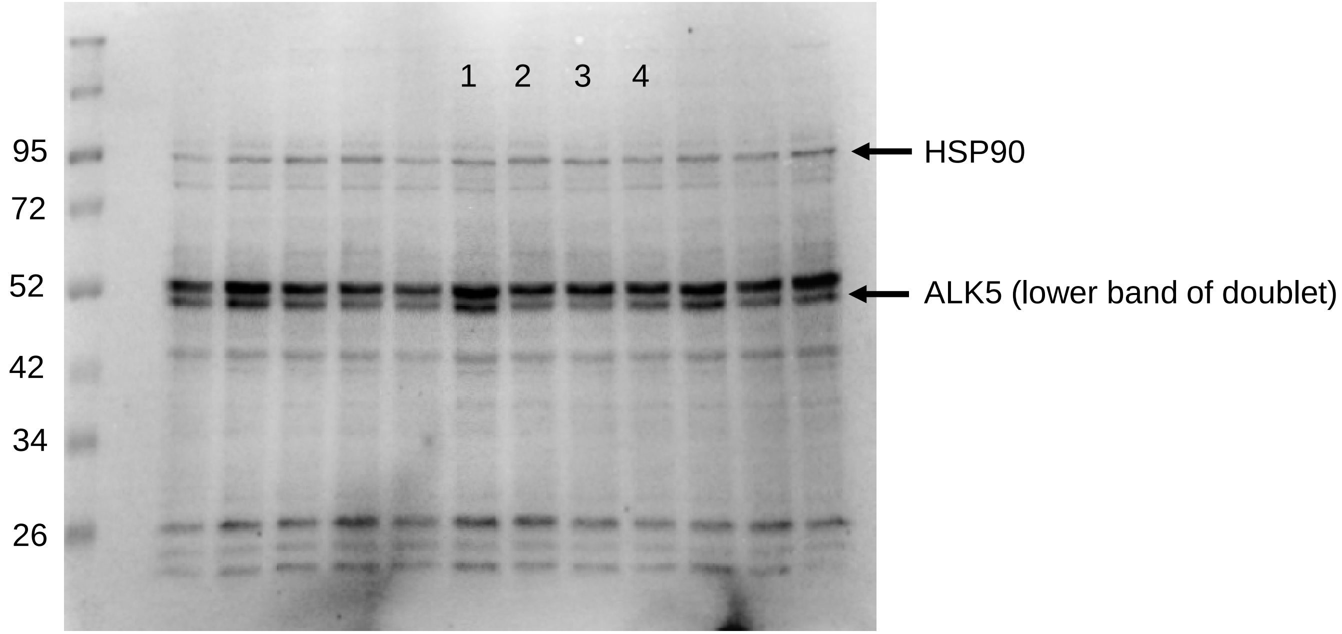

Immunoblot Figure 1C

Densitometric values: 5596812 3265197 2898636 3421590

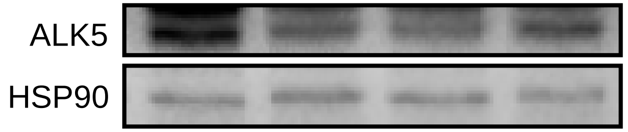

Densitometric values: 2876536 2572644 2966908 2638094

Ratio: 1.95 1.27 0.98 1.30

Immunoblot Supplementary Figure S1C

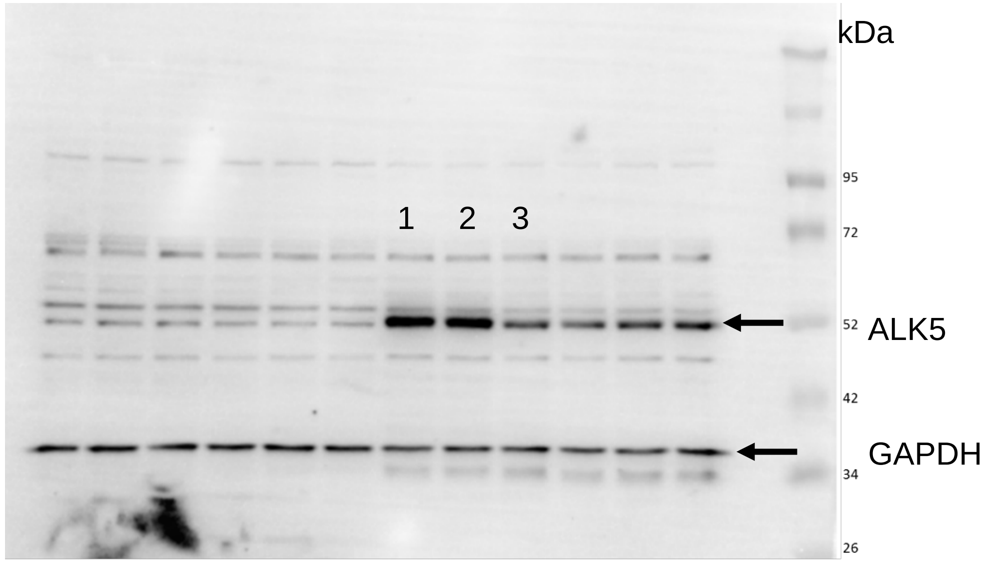

Immunoblot Supplementary Figure S1C

Densitometric values: 10478332 10266431 4839960

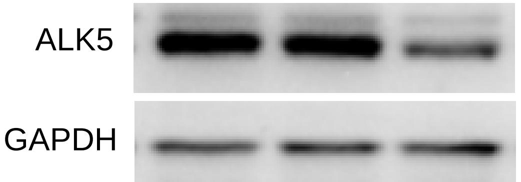

Densitometric values: 3824209 3845105 4136718

Ratio: 2.74 2.67 1.17

Supplementary Material: Full blots and band quantification: Figure 2B

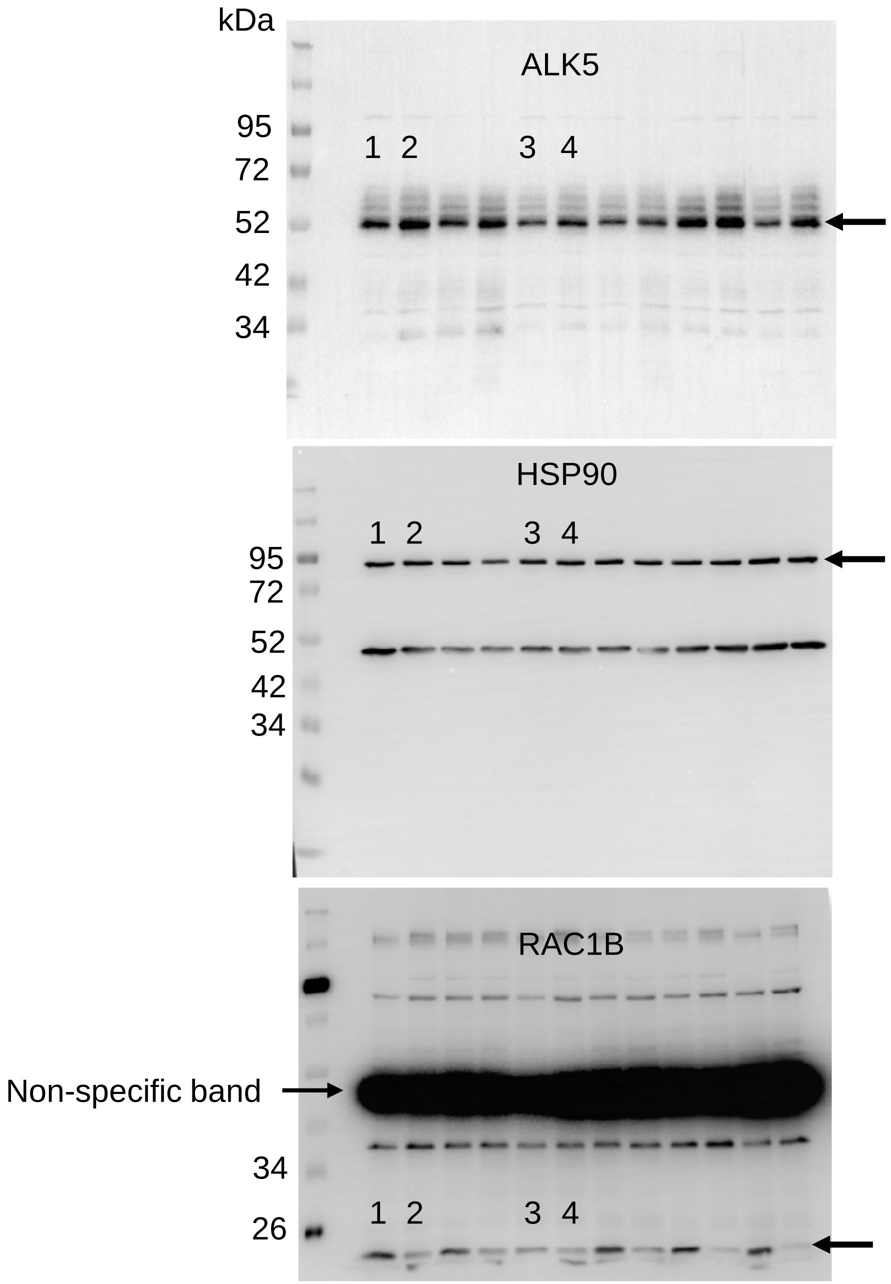

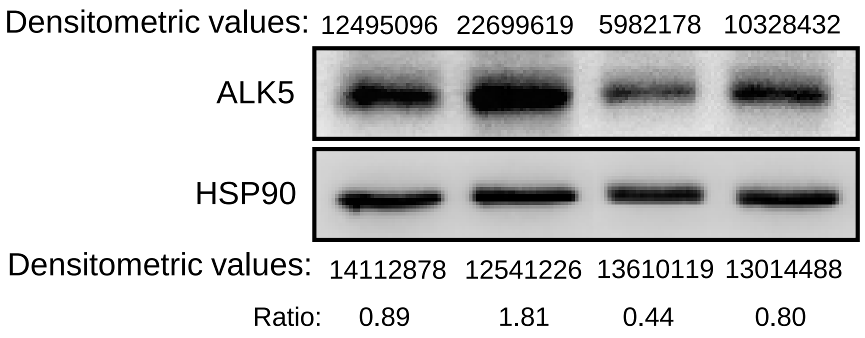

Supplementary Material: Full blots and band quantification: Figure 2E

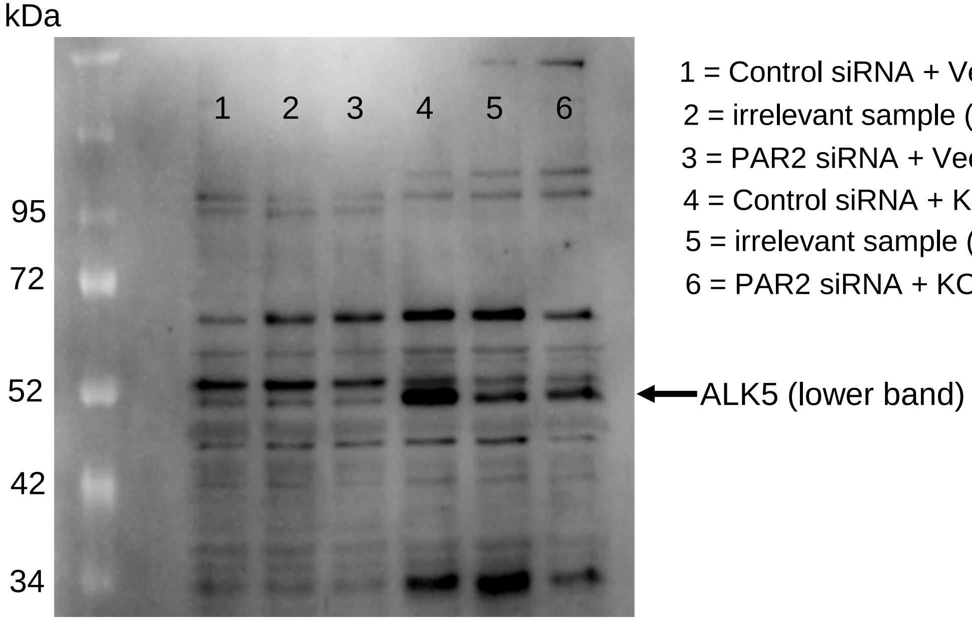

1 = Control siRNA + Vector
2 = irrelevant sample (removed)
3 = PAR2 siRNA + Vector
4 = Control siRNA + KO
5 = irrelevant sample (removed)
6 = PAR2 siRNA + KO

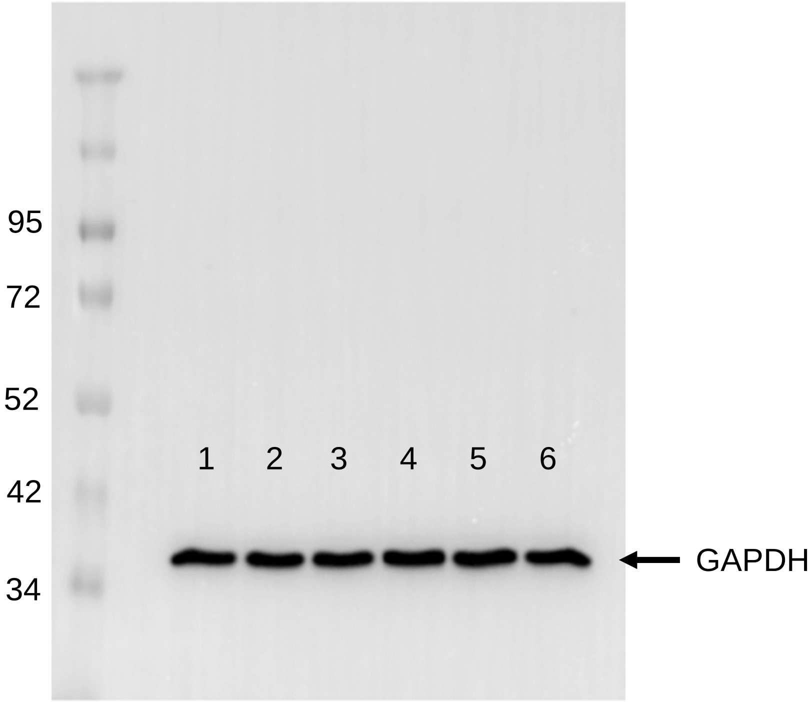

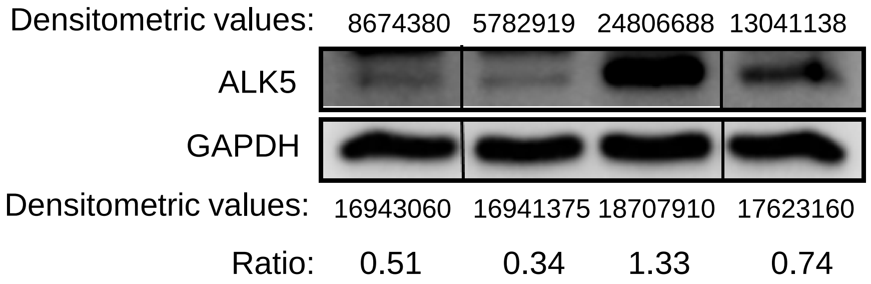

Supplementary Material: Full blots and band quantification: Figure 4B

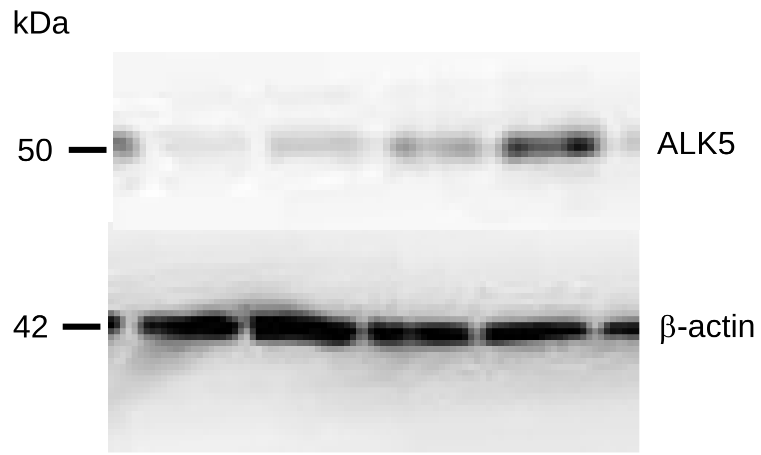

Supplement: Supplementary file 1 [file cancers-11-01211-s001.pdf]
